# Costing of AMR can Incentivize Responses to One Health

**DOI:** 10.5334/aogh.5355

**Published:** 2026-06-10

**Authors:** Pushpam Kumar

**Affiliations:** 1 Visiting Fellow, Boston College, Boston and former Chief Environmental Economist, UNEP, Boston, USA

**Keywords:** economics of antimicrobial resistence

## Abstract

Monetising the damage inflicted to economy and society is necessary and it is doable. The costing of antimicrobial resistance (AMR) enables clearer policy response and removes haziness in decisions. Costing the AMR facilitates the investment in interventions, needed for implementation of One Health.

The damage done to human and other animal health by developing resistance against drugs is serious and significant in an interconnected world. The costs associated with antimicrobial resistance (AMR) are one of the most devastating phenomena of our time. AMR could result in US$ 1 trillion of additional healthcare costs per year by 2050 and US$ 1 trillion to 3.4 trillion of gross domestic product losses per year by 2030 [1]. One can see the associated loss of human capital.

Let us consider the magnitude of the problem arising from AMR. In 2019, 4.95 million deaths were associated with drug-resistant bacterial infections, including 1.27 million deaths directly attributable to bacterial AMR; without an adequate intervention, the life expectancy could decline by 1.8 years by 2035 due to AMR [2].

Most of these numbers are missing from the transaction and planning of the governments and business exchanges of the private sectors. This is most alarming and leads to the situation where the necessary investment to curb the menace of AMR is either insufficient or missing altogether. The costing and finding of damages inflicted by the AMR is critically important for harnessing the human capital and eventually for the well-being and sustainability of the nations.

It is not surprising that in 2024, the UN General Assembly **called upon the Quadripartite secretariat (Food and Agriculture Organization [FAO], World Health Organization [WHO], United Nations Environment Program [UNEP], and World Organization for Animal Health [WOAH]) to** “**facilitate the AMR investment cases** in collaboration with relevant stakeholders to facilitate the mobilization of domestic and external financing for AMR response across all sectors in LMICs.”

I, as the Advisory Board Member of the Quadripartite Joint Secretariat (QJS) on AMR, designed and conducted the economic case for the investment in intervention targeted at AMR found this exercise challenging but extremely useful. Increased morbidity and mortality from drug-resistant infections will lead to lower workforce participation and productivity losses of US$ 443 billion per year.

This is serious. Any intervention scheme to combat the AMR would cost a lot less than the benefits of avoiding costs and therefore we have an adequate rationale for undertaking this investment. It must be mentioned here that a large number of indirect costs of AMR, like impact on animal health, horticulture, and noncommunicable diseases like mental health, remained outside the calculus [3]. And that further enhances the return on investment in AMR intervention.

The other well-accepted global agenda of One Health where the human, planetary, and animal health are intertwined and are popularly known as One Health will further benefit if the blended finance is trapped in the AMR management. If implemented globally, AMR interventions across sectors set out in the study are expected to cost an average of US$ 46 billion per year and bring a return of between US$ 7 and US$ 13 for every US$ 1 spent by 2050. Evaluation of a mixed policy of One Health interventions—including awareness raising, surveillance, optimizing antimicrobial use in human and animal health, infection prevention, better management of effluent, and new treatments—is estimated to cost US$ 1420.7 billion worldwide by 2050, or US$ 45.8 billion per year.

These costs directly and adversely affect the sustainability of economy. The economies and their sustainability, especially in the countries of the South, rely more on Human Capital than on produced or natural capital. For example, the latest Inclusive Wealth for Africa provides an estimate where more than half of the wealth comprises human capital. Therefore, an estimate on a regular basis is needed to keep track of sustainability. But more than this, we can proxy this number to benefits of intervention against AMR, by preventing the costs.

The costing of AMR, on the one hand, would incentivize the investment in AMR and would provide authoritative accounts to make investment in One Health Agenda as lucrative and not merely an unproductive spending.

Various solutions can prevent, halt, or fight AMR at different stages. No single solution exists, rather, the combination of these options is the only effective action against the negative impacts of AMR. What is important here is to recognize some fundamental insights emerging from the exercise of economic case for the AMR. First, the costs related to AMR are tangible and with some input from epidemiologists and economists it can be computed with high level of accuracy. Second, these costs are spread out across large populations over many years, they hardly influence fiscal and monetary calculations of national budgets and are buried deep in healthcare costs and in lost productivity. Third, most of the intervention costs are in the initial stages while the benefits (avoidance of costs across different populations and sectors) are spread over time, sometime very far. That requires a judicious choice of discount rate (probably lower than the prevailing one and used by the project managers). Fourth, the costs and benefits of AMR must be put into a larger perspective of financial planning and portfolio choices. Arguments based on emotions would not help, hard numbers will. Fifth, economics of AMR requires a strong Community of Practitioners who can share challenges and knowledge gaps in data, methodological innovation, conflicting choices, and prevailing tradeoffs.

Everyone is aware of the challenges in reliable health data, capacity constraints, and biases of all types when dealing with the invisible costs of AMR, but our role should be to organize the challenges rather than ignore them. Issues like optimizing the use of antimicrobial medicines in human and animal health are the need of our time but developing the economic case for sustainable investment in new medicines, diagnostic tools, vaccines, and other interventions for the AMR would help us in achieving the SDGs and the well-being of people.
